# Association of Coronary Artery Calcium With Long-term, Cause-Specific Mortality Among Young Adults

**DOI:** 10.1001/jamanetworkopen.2019.7440

**Published:** 2019-07-19

**Authors:** Michael D. Miedema, Zeina A. Dardari, Khurram Nasir, Ron Blankstein, Thomas Knickelbine, Sandra Oberembt, Leslee Shaw, John Rumberger, Erin D. Michos, Alan Rozanski, Daniel S. Berman, Matthew J. Budoff, Michael J. Blaha

**Affiliations:** 1 Minneapolis Heart Institute Foundation, Minneapolis, Minnesota; 2 Ciccarone Center for the Prevention of Cardiovascular Disease, Division of Cardiology, Department of Medicine, Johns Hopkins School of Medicine, Baltimore, Maryland; 3 Center for Prevention and Wellness Research, Baptist Health Medical Group, Miami Beach, Florida; 4 Department of Medicine, Cardiovascular Division, Brigham and Women’s Hospital, Boston, Massachusetts; 5 Department of Radiology, Brigham and Women’s Hospital, Boston, Massachusetts; 6 Department of Radiology, Weill Cornell Medical College, New York, New York; 7 Department of Cardiac Imaging, The Princeton Longevity Center, Princeton, New Jersey; 8 Department of Medicine, Mount Sinai, New York, New York; 9 Department of Imaging, Smidt Heart Institute, Cedars-Sinai, Los Angeles, California; 10 Department of Medicine, Smidt Heart Institute, Cedars-Sinai, Los Angeles, California; 11 Los Angeles BioMedical Research Institute, Harbor University of California Los Angeles Medical Center, Torrance

## Abstract

**Importance:**

The level of coronary artery calcium (CAC) can effectively stratify cardiovascular risk in middle-aged and older adults, but its utility for young adults is unclear.

**Objectives:**

To determine the prevalence of CAC in adults aged 30 to 49 years and the subsequent association of CAC with coronary heart disease (CHD), cardiovascular disease (CVD), and all-cause mortality.

**Design, Setting, and Participants:**

A multicenter retrospective cohort study was conducted among 22 346 individuals from the CAC Consortium who underwent CAC testing (baseline examination, 1991-2010, with follow-up through June 30, 2014; CAC quantified using nonconrast, cardiac-gated computed tomography scans) for clinical indications and were followed up for cause-specific mortality. Participants were free of clinical CVD at baseline. Statistical analysis was performed from June 1, 2017, to May 31, 2018.

**Main Outcomes and Measures:**

The prevalence of CAC and the subsequent rates of CHD, CVD, and all-cause mortality. Competing risks regression modeling was used to calculate multivariable-adjusted subdistribution hazard ratios for CHD and CVD mortality.

**Results:**

The sample of 22 346 participants (25.0% women and 75.0% men; mean [SD] age, 43.5 [4.5] years) had a high prevalence of hyperlipidemia (49.6%) and family history of CHD (49.3%) but a low prevalence of current smoking (11.0%) and diabetes (3.9%). The prevalence of any CAC was 34.4%, with 7.2% having a CAC score of more than 100. During follow-up (mean [SD], 12.7 [4.0] years), there were 40 deaths related to CHD, 84 deaths related to CVD, and 298 total deaths. A total of 27 deaths related to CHD (67.5%) occurred among individuals with CAC at baseline. The CHD mortality rate per 1000 person-years was 10-fold higher among those with a CAC score of more than 100 (0.69; 95% CI, 0.41-1.16) compared with those with a CAC score of 0 (0.07; 95% CI, 0.04-0.12). After multivariable adjustment, those with a CAC score of more than 100 had a significantly increased risk of CHD (subdistribution hazard ratio, 5.6; 95% CI, 2.5-12.7), CVD (subdistribution hazard ratio, 3.3; 95% CI, 1.8-6.2), and all-cause mortality (hazard ratio, 2.6; 95% CI, 1.9-3.6) compared with those with a CAC score of 0.

**Conclusions and Relevance:**

In a large sample of young adults undergoing CAC testing for clinical indications, 34.4% had CAC, and those with elevated CAC scores had significantly higher rates of CHD and CVD mortality. Coronary artery calcium may have potential utility for clinical decision-making among select young adults at elevated risk of cardiovascular disease.

## Key Points

**Question:**

What is the prevalence of coronary artery calcium (CAC) in adults aged 30 to 49 years with clinical indications for CAC scoring, and is CAC associated with long-term, cause-specific mortality in these young adults?

**Findings:**

In this cohort study of 22 346 individuals from the CAC Consortium with clinical indications for CAC, 34.4% had prevalent CAC. The risk of death from coronary heart disease, cardiovascular disease, or all-cause mortality was significantly higher for those with elevated CAC scores, even after multivariable adjustment.

**Meaning:**

Coronary artery calcium may potentially be used as a tool to aid decision-making among select young adults at elevated lifetime risk for cardiovascular disease; the relatively high prevalence of CAC in younger adults with cardiovascular risk factors reinforces the need for the adoption of healthy lifestyle behaviors early in life.

## Teaser

This cohort study uses data from the CAC Consortium to examine the prevalence of coronary artery calcium in young adults and the subsequent association of coronary artery calcium with coronary heart disease, cardiovascular disease, and all-cause mortality.

## Introduction

Multiple current cardiovascular guidelines rely on estimates of 10-year absolute risk of cardiovascular disease (CVD) to guide decisions regarding the allocation of preventive CVD medications.^1,2,3,4^ Coronary heart disease (CHD) and CVD risk equations are heavily driven by age; as a result, younger adults are typically estimated to have a low 10-year risk of CVD despite the presence of nonoptimal risk factors and an elevated lifetime risk of CVD.^5^ Experts have suggested that earlier treatment for young adults holds the potential to regress and suppress early atherosclerosis, although who to treat and when to treat remain unclear.^6^

Coronary artery calcium (CAC) is a direct marker of atherosclerosis that can robustly stratify risk for individuals without known CVD,^7^ allowing it to serve as a tool to aid clinical decision-making regarding preventive therapies for middle-aged adults.^8,9,10,11,12^ The utility of CAC in younger populations is less clear. Prior CAC studies for younger adults have been limited by small sample sizes, a short duration of follow-up, and a lack of cause-specific mortality.^13,14,15^ In response to calls for further research on this subject,^16^ we sought to provide data from the CAC Consortium to help determine what role, if any, CAC may play in the identification of young adults at higher risk for CVD who may be candidates for more aggressive therapy for CVD prevention.

## Methods

### Study Design and Study Population

The CAC Consortium is an investigator-initiated, multicenter retrospective cohort comprising 66 636 patients that is aimed at determining the association of CAC with long-term, cause-specific mortality. The rationale, methods, and baseline results of the cohort have been previously published.^17^ In brief, the CAC Consortium consists of patients from 4 high-volume centers from 3 states (California, Minnesota, and Ohio) with long-standing experience in CAC scoring and with detailed patient data on demographics, risk factors, and CAC score results. All patients were at least 18 years of age and free of clinical CVD or cardiovascular symptoms at the time of CAC testing. Participants were screened for cardiovascular symptoms and excluded if they were symptomatic at baseline. Clinical indications for CAC testing include individuals with CVD risk factor(s) who are uncertain about their absolute risk of CVD. Clinical indications were not specifically documented, but most participants underwent CAC testing owing to either hyperlipidemia or a family history of CVD. Written informed consent for participation in research was obtained at the individual centers at baseline, and institutional review board approval for coordinating center activities, including death ascertainment and death certificate collection, was obtained at Johns Hopkins University School of Medicine. This study followed the Strengthening the Reporting of Observational Studies in Epidemiology (STROBE) reporting guidelines for cohort studies.

Baseline data for the CAC Consortium represent the years 1991 through 2010, with follow-up through June 30, 2014. Comparison of the CAC Consortium with the National Health and Nutrition Examination Survey 2001-2002, the Multi-Ethnic Study of Atherosclerosis, and the Framingham Offspring Study have been previously published,^17^ suggesting comparable baseline characteristics, use of pharmacotherapy, and mortality rates with these contemporary cohorts. For this study, we excluded 43 979 patients 50 years or older and 311 patients younger than 30 years, leaving 22 346 for the analysis.

### Definition of Cardiovascular Risk Factors

Data on risk factors and laboratory test results were collected as part of the routine clinical visit associated with the referral for CAC testing and/or from a semistructured in-person interview at the time of the CAC scan. Hypertension and diabetes were defined by a prior clinical diagnosis or treatment with antihypertensive therapy or glucose-lowering therapy. Dyslipidemia was defined as a prior diagnosis of primary hyperlipidemia (low-density lipoprotein cholesterol level >160 mg/dL [to convert to millimoles per liter, multiply by 0.0259]), dyslipidemia (elevated triglycerides level >150 mg/dL [to convert to millimoles per liter, multiply by 0.0113] and/or low high-density lipoprotein cholesterol level <40 mg/dL in men and <50 mg/dL in women [to convert to millimoles per liter, multiply by 0.0259]), or treatment with any lipid-lowering drug. Smoking was based on self-report and, for this analysis, was defined as current smoking or no current smoking. Family history of CHD was predominantly determined by the presence of a first-degree relative with a history of CHD at any age.

The 10-year risk of atherosclerotic CVD (ASCVD) was calculated according to the Pooled Cohort Equations (PCE) calculator.^1^ The PCE is intended for individuals aged 40 to 79 years. For individuals aged 30 to 39 years, we used the raw PCE equations with removal of the age limitation and calculated a mean (SD) 10-year risk of 1.1% (1.3%), with a weighted mean 10-year risk of 2.2% (2.0%) for the entire sample. Lifetime risk of CVD was estimated using the risk algorithm from a previously published meta-analysis.^18^

To account for partially missing risk factor data (27.8% of the cohort had missing data, although most were missing only 1 risk factor), multiple imputation was conducted leveraging nonmissing age, sex, race/ethnicity, and nonmissing risk factor data and the CAC score. The details of the imputation method and validation of this approach have previously been described.^17^ In summary, the validation of the imputation approach showed nearly identical mean and median ASCVD risk scores and a robust correlation coefficient of 0.952 between the imputed and directly calculated ASCVD scores.

### CAC Testing

Coronary artery calcium was quantified using noncontrast, cardiac-gated computed tomography scans at each site according to the common standard protocol for each computed tomography scanner technology. The details of the computed tomography scanners used at each site have been previously published.^17^ Coronary artery calcium was quantified for all participants via the Agatston method, with CAC score categories of 0, 1 to 100, and higher than 100. Coronary artery calcium scores provide quantification of the total plaque burden in the coronary arteries, with higher scores indicating a larger burden of plaque and subsequently a higher risk for ASCVD events.

### Outcome Ascertainment

The primary outcomes for this study were CHD, CVD, and total mortality during a mean (SD) of 12.7 (4.0) years of follow-up. Mortality was assessed in the CAC Consortium via the linkage of patient records with the Social Security Administration Death Master File using a previously validated algorithm.^19^ The algorithm uses unique patient identifiers in a semiflexible hierarchical matching process similar to the algorithm used by the National Death Index service. Death certificates were obtained from the National Death Index service, and the underlying cause of death was categorized into common causes of death using *International Classification of Diseases, Ninth Revision* and *International Statistical Classification of Diseases and Related Health Problems, Tenth Revision* codes as previously described.^17^ Internal validation studies against known deaths identified via the electronic medical record revealed greater than 90% specificity for identifying known deaths, with sensitivity between 72% and 90%.

### Statistical Analysis

Statistical analysis was performed from June 1, 2017, to May 31, 2018. Baseline characteristics are presented for the total sample and stratified by sex. Proportions are presented for categorical variables plus means with SDs or medians with interquartile ranges depending on the normality of the data. χ^2^ Testing and analysis of variance or Kruskal-Wallis testing were used for formal comparisons, as appropriate. Absolute mortality rates according to CAC categories are expressed per 1000 person-years. All *P* values were from 2-sided tests, and results were deemed statistically significant at *P* < .05.

Competing risks regression was used to estimate risk of cause-specific mortality (with non-CHD–related or non-CVD–related death used as the competing cause); thus, results were reported as subdistribution hazard ratios with 95% CIs, while Cox proportional hazards regression was used to estimate hazard ratios for the association between CAC categories and all-cause mortality. Schoenfeld residuals were used to confirm the proportional hazards assumption, and no violation was found.

Additional subgroup analyses were performed after stratifying by age, including age groups 30 to 39 years and 40 to 49 years, and lifetime risk, with grouping by optimal or nonoptimal risk factors (low lifetime risk) and elevated or major risk factors (high lifetime risk) as described by Berry et al.^18^ All analyses were performed with Stata software, version 14.2 (StataCorp).

## Results

### Baseline Demographics, CAC Prevalence, and Subsequent Mortality

The baseline characteristics and 10-year estimated ASCVD risk of the 22 346 individuals included in the study (25.0% women and 75.0% men; mean [SD] age, 43.5 [4.5] years; 12 007 of 13 696 individuals [87.7%] of white race/ethnicity) are shown in Table 1 both for the total sample and stratified by sex. The prevalence of hyperlipidemia (49.6%) and the prevalence of a family history of CHD (49.3%) were high, while the prevalence of current smoking (11.0%) and the prevalence of diabetes (3.9%) were relatively low. The mean (SD) 10-year risk of ASCVD for the total sample was 2.2% (2.0%), with 92.7% of the sample having a 10-year risk of ASCVD of less than 5%.

**Table 1.** Baseline Characteristics and Estimated 10-Year CVD Risk Among 22 346 Asymptomatic Individuals Aged 30 to 49 Years From the CAC Consortium

Characteristic	Individuals, No. (%)			*P* Value
	All (N = 22 346)	Women (n = 5576)	Men (n = 16 770)	
Age, mean (SD), y	43.5 (4.5)	44.0 (4.4)	43.3 (4.6)	<.001
Race/ethnicity, No./total No. (%)				
White	12 007/13 696 (87.7)	3123/3657 (85.4)	8884/10 039 (88.5)	<.001
Asian	591/13 696 (4.3)	181/3657 (4.9)	410/10 039 (4.1)	
Black	316/13 696 (2.3)	114/3657 (3.1)	202/10 039 (2.0)	
Hispanic	520/13 696 (3.8)	175/3657 (4.8)	345/10 039 (3.4)	
Other	262/13 696 (1.9)	64/3657 (1.8)	198/10 039 (2.0)	
Current smoker	2466 (11.0)	646 (11.6)	1820 (10.9)	.13
Hypertension	4496 (20.1)	1045 (18.7)	3451 (20.6)	.003
Hyperlipidemia	11 082 (49.6)	2346 (42.1)	8736 (52.1)	<.001
Diabetes	882 (3.9)	236 (4.2)	646 (3.9)	.21
Family history of CHD	11 006 (49.3)	3123 (56.0)	7883 (47.0)	<.001
CVD risk, mean (SD)				
Lifetime	42 (9.0)	34 (7.0)	44 (9.0)	<.001
10 y^a^	2.2 (2.0)	1.3 (1.3)	2.5 (2.2)	<.001
10-y categories				
<5%	20 709 (92.7)	5454 (97.8)	15 255 (91.0)	<.001
5%-7.5%	1049 (4.7)	90 (1.6)	959 (5.7)	
>7.5%	588 (2.6)	32 (0.6)	556 (3.3)	

Abbreviations: CAC, coronary artery calcium; CHD, coronary heart disease; CVD, cardiovascular disease.

^a^ The 10-year risk was calculated using the Pooled Cohort Equations.

For the total sample, the prevalence of any CAC was 34.4% (n = 7686; the number needed to screen to detect any CAC was 3), while 7.2% (n = 1606; the number needed to screen to detect any CAC was 14) had a CAC score higher than 100 (Table 2). The prevalence of any CAC across the age range in our study stratified by sex is shown in the eFigure in the Supplement.

**Table 2.** Prevalence of CAC and Subsequent Mortality Among 22 346 Asymptomatic Individuals Aged 30 to 49 Years From the CAC Consortium

Variable	All (N = 22 346)	Women (n = 5576)	Men (n = 16 770)	*P* Value
CAC prevalence, No. (%)^a^				
0	14 660 (65.6)	4613 (82.7)	10 047 (59.9)	<.001
1-100	6080 (27.2)	831 (14.9)	5249 (31.3)	
>100	1606 (7.2)	132 (2.4)	1474 (8.8)	
CHD-related death				
No. (%)	40 (0.2)	2 (0.04)	38 (0.2)	.004
Rate (95% CI)^b^	0.14 (0.10-0.19)	0.03 (0.01-0.12)	0.18 (0.13-0.24)	.004
CVD-related death				
No. (%)	84 (0.4)	12 (0.2)	72 (0.4)	.02
Rate (95% CI)^b^	0.30 (0.24-0.37)	0.17 (0.10-0.31)	0.34 (0.27-0.42)	.03
All-cause death				
No. (%)	298 (1.3)	66 (1.1)	232 (1.4)	.26
Rate (95% CI)^b^	1.10 (0.94-1.12)	0.96 (0.75-1.21)	1.10 (0.95-1.20)	.35
Cause of death, No./total No. (%)				
Neoplasms	86/298 (28.9)	33/66 (50.0)	53/232 (22.8)	.001
CVD	84/298 (28.2)	12/66 (18.2)	72/232 (31.0)	
Injury and poisoning	42/298 (14.6)	6/66 (9.1)	36/232 (15.5)	

Abbreviations: CAC, coronary artery calcium; CHD, coronary heart disease; CVD, cardiovascular disease.

^a^ The CAC prevalence was categorized according to Agatston scores of 0, 1 to 100, and higher than 100.

^b^ Rate per 1000 person-years.

During the mean (SD) 12.7 (4.0) years of follow-up (range of maximum follow-up across the 4 sites, 13.6-22.5 years), there were 40 deaths related to CHD, 84 deaths related to CVD, and 298 total deaths (1.3% of the sample), with an all-cause mortality rate of 1.10 (95% CI, 0.94-1.12) deaths per 1000 person-years (Table 2). The top 2 causes of death were cancer (86 [28.9%]) and CVD (84 [28.2%]). The rate of all-cause mortality was similar between the sexes (women, 0.96; 95% CI, 0.75-1.21; men, 1.10; 95% CI, 0.95-1.20; *P* = .35), while men had higher rates than women of CVD mortality (0.34; 95% CI, 0.27-0.42 vs 0.17; 95% CI, 0.10-0.31; *P* = .03) and CHD mortality (0.18; 95% CI, 0.13-0.24 vs 0.03; 95% CI, 0.01-0.12; *P* = .004).

The prevalence of traditional CVD risk factors and subsequent mortality rates according to baseline CAC scores are shown in Table 3. The prevalence for each of the 5 traditional risk factors (hypertension, hyperlipidemia, current smoking, family history of CHD, and diabetes) was significantly increased in those with a CAC score of more than 100 compared with those with a CAC score of 0. However, 662 of the 1606 individuals (41.2%) with a CAC score higher than 100 had 0 or 1 traditional risk factors. The rate of CHD mortality per 1000 person-years was 4-fold higher among individuals with any CAC (0.27 CHD-related deaths per 1000 person-years; 95% CI, 0.19-0.40) and 10-fold higher among individuals with a CAC score higher than 100 (0.69 CHD-related deaths per 1000 person-years; 95% CI, 0.41-1.16) compared with individuals with a CAC score of 0 (0.07 CHD-related deaths per 1000 person-years; 95% CI, 0.04-0.12). A total of 27 of 40 CHD-related deaths (67.5%) occurred among individuals with CAC at baseline.

**Table 3.** Prevalence of Traditional CVD Risk Factors and Subsequent Mortality According to Baseline CAC Score Among 22 346 Asymptomatic Individuals Aged 30 to 49 Years From the CAC Consortium

CVD Risk Factor	Individuals, No. (%)			*P* Value
	CAC Score = 0 (n = 14 660 [65.6%])^a^	CAC Score = 1-100 (n = 6080 [27.2%])	CAC Score >100 (n = 1606 [7.2%])	
Hypertension	2567 (17.5)	1435 (23.6)	494 (30.8)	<.001
Hyperlipidemia	6684 (45.6)	3401 (55.9)	997 (62.1)	<.001
Current smoker	1521 (10.4)	706 (11.6)	239 (14.9)	<.001
Family history of CHD	6947 (47.4)	3150 (51.8)	909 (56.6)	<.001
Diabetes	450 (3.1)	296 (4.9)	136 (8.5)	<.001
Total No. of risk factors				
0	3389 (23.1)	975 (16.0)	176 (11.0)	<.001
1	5819 (39.7)	2204 (36.3)	486 (30.3)	
≥2	4159 (28.4)	2040 (33.6)	605 (37.7)	
**Mortality**				
CHD-related death				
No. (%)	13 (0.09)	13 (0.2)	14 (0.9)	<.001
Rate (95% CI)^b^	0.07 (0.04-0.12)	0.17 (0.10-0.29)	0.69 (0.41-1.16)	
CVD-related death				
No. (%)	36 (0.2)	28 (0.5)	20 (1.2)	<.001
Rate (95% CI)^b^	0.20 (0.14-0.27)	0.36 (0.25-0.52)	0.98 (0.63-1.51)	
All-cause death				
No. (%)	151 (1.0)	90 (1.5)	57 (3.5)	<.001
Rate (95% CI)^b^	0.82 (0.70-0.96)	1.23 (0.94-1.40)	2.81 (2.22-3.63)	

Abbreviations: CAC, coronary artery calcium; CHD, coronary heart disease; CVD, cardiovascular disease.

^a^ The CAC prevalence was categorized according to Agatston scores of 0, 1 to 100, and higher than 100.

^b^ Rate per 1000 person-years.

Subdistribution hazard ratios for CHD and CVD and hazard ratios for all-cause mortality according to subgroups of CAC are shown in Table 4. After adjustment for traditional risk factors, the risk of death due to CHD remained significantly increased among individuals with a CAC score higher than 100 compared with those with a CAC score of 0 (subdistribution hazard ratio, 5.6; 95% CI, 2.5-12.7), as did the risk of CVD mortality (subdistribution hazard ratio, 3.3; 95% CI, 1.8-6.2) and all-cause mortality (hazard ratio, 2.6; 95% CI, 1.9-3.6).

**Table 4.** Subdistribution Hazard Ratios for CHD and CVD and Hazard Ratios for All-Cause Mortality Among 22 346 Asymptomatic Individuals Aged 30 to 49 Years From the CAC Consortium

CAC Prevalence^a^	Hazard Ratio (95% CI)		
	Unadjusted	Model 1^b^	Model 2^c^
CHD mortality			
CAC score			
0	1 [Reference]	1 [Reference]	1 [Reference]
1-100	2.4 (1.1-5.1)	2.0 (0.9-4.4)	1.7 (0.8-3.9)
>100	9.7 (4.5-20.6)	7.8 (3.4-17.9)	5.6 (2.5-12.7)
CVD mortality			
CAC score			
0	1 [Reference]	1 [Reference]	1 [Reference]
1-100	1.8 (1.1-3.0)	1.7 (1.0-2.9)	1.5 (0.9-2.5)
>100	5.0 (2.9-8.7)	4.5 (2.5-8.4)	3.3 (1.8-6.2)
All-cause mortality			
CAC score			
0	1 [Reference]	1 [Reference]	1 [Reference]
1-100	1.4 (1.1-1.8)	1.4 (1.0-1.8)	1.2 (0.9-1.6)
>100	3.4 (2.5-4.7)	3.1 (2.3-4.3)	2.6 (1.9-3.6)

Abbreviations: CAC, coronary artery calcium; CHD, coronary heart disease; CVD, cardiovascular disease.

^a^ The CAC prevalence was categorized according to Agatston scores of 0, 1 to 100, and higher than 100.

^b^ Adjusted for age and sex.

^c^ Adjusted for age, sex, hyperlipidemia, hypertension, smoking, diabetes, and a family history of CHD.

Of the 22 346 individuals in our sample, 14 233 (63.7%) were found to have elevated and/or major CVD risk factors and were categorized as having a high lifetime risk for CVD. Of the 40 deaths related to CHD during follow-up, 34 (85.0%) occurred among individuals with an elevated lifetime risk for CVD. After multivariable adjustment, a significant association remained between CAC and the risk for CHD, CVD, and all-cause mortality among individuals with a high lifetime risk for CVD (eTable in the Supplement). A similar association was seen between CAC and mortality risk among individuals with a low lifetime risk for CVD, although the association between CAC and CHD risk did not reach statistical significance, likely owing to the low number of CHD-related deaths in this group.

Finally, to further refine our results by baseline age, we conducted additional analyses stratifying by age groups of 30 to 39 years and 40 to 49 years (Figure). The prevalence of CAC was significantly higher in individuals aged 40 to 49 years compared with those aged 30 to 39 years, with 29.3% of individuals aged 40 to 49 years having a CAC score of 1 to 100 and 8.4% of individuals aged 40 to 49 years having a CAC score higher than 100 and with 19.1% of individuals aged 30 to 39 years having a CAC score of 1 to 100 and 2.7% of individuals aged 30 to 39 years having a CAC score of more than 100. There was a graded increase in CHD, CVD, and all-cause mortality across increasing CAC strata for individuals aged 30 to 39 years and for those aged 40 to 49 years. Although individuals aged 30 to 39 years were less likely to have CAC, mortality rates for those with a CAC score higher than 100 appeared to be similar between those aged 30 to 39 years and those aged 40 to 49 years.

**Figure.**
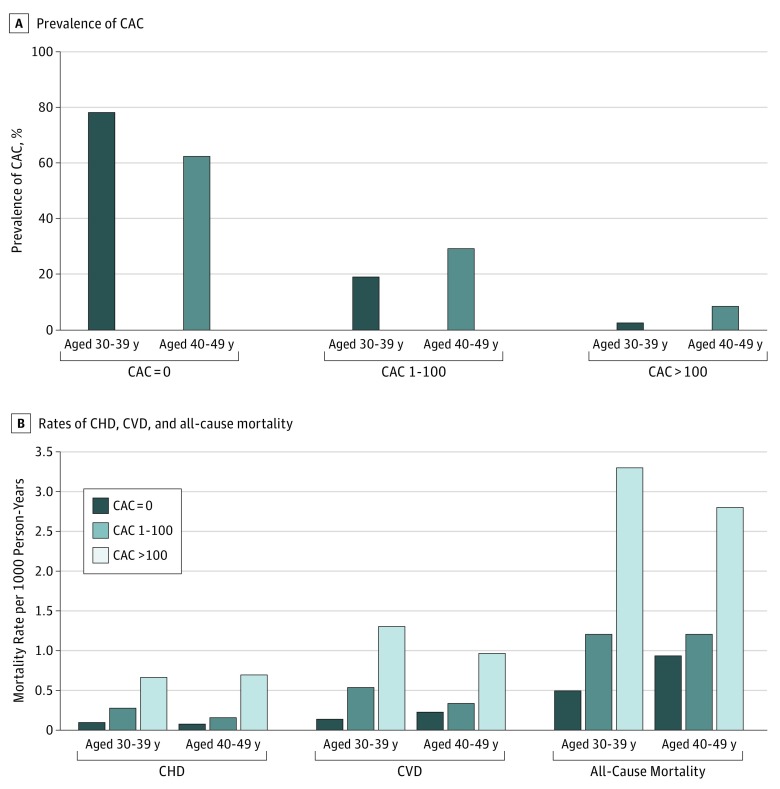
Prevalence of Coronary Artery Calcium (CAC) and Subsequent Rates of Coronary Heart Disease (CHD), Cardiovascular Disease (CVD), and All-Cause Mortality Among Young Adults A, Prevalence of CAC in 4544 asymptomatic individuals aged 30 to 39 years and 17 802 individuals aged 40 to 49 years enrolled in the CAC Consortium stratified by baseline CAC. B, Subsequent rates of CHD, CVD, and all-cause mortality. The CAC prevalence was categorized according to Agatston scores.

## Discussion

In a large, multicenter cohort with long-term follow-up of adults aged 30 to 49 years undergoing CAC testing for clinical indications, we found that 34.4% of the sample had any CAC and that 7.2% had significantly elevated CAC scores (>100). Subsequent rates of CHD, CVD, and total mortality were significantly elevated among individuals with elevated CAC, with 67.5% of all future CHD-related deaths occurring among those with any baseline CAC. After multivariable adjustment, there was more than a 5-fold increase in risk for CHD mortality and a 3-fold increase in risk for CVD mortality among individuals with a CAC score higher than 100 compared with those with a CAC score of 0. The high prevalence of premature coronary atherosclerosis in this sample of younger adults highlights the need to increase focus on the importance of adopting healthy lifestyle behaviors early in life. In addition, these findings suggest that CAC testing could potentially be considered for further risk stratification in select young adults with elevated cardiovascular risk.

### Comparisons With Prior Data

In general, data on CAC in young adults are limited. A prior study from a separate referral-based cohort found that, among 8143 individuals younger than 45 years at the time of CAC screening (mean age, 40 years), approximately 30% had CAC, and the risk of all-cause mortality increased across categories of CAC.^15^ The study was limited by a short duration of follow-up (mean, 5.8 years) and a lack of cause-specific mortality. Our results found a similar graded increase in rates of all-cause mortality across categories of CAC, although our overall mortality rates were lower, potentially owing to the more contemporary nature of our data and the lower population rates of CHD- and CVD-related deaths compared with data from prior decades.^20^

A recent analysis from the community-based Coronary Artery Risk Development in Young Adults (CARDIA) prospective cohort study found that approximately 10% of the 3043 CARDIA participants aged 32 to 46 years (mean age, 40.3 years) had any CAC and that those individuals had a subsequent 5-fold higher risk for incident CHD events and a 3-fold higher risk for incident CVD events during 12.5 years of follow-up.^14^ The CARDIA study was limited by a relatively small sample size with very few CHD- and CVD-related deaths but did demonstrate that even low CAC scores among young adults are associated with a higher risk of CHD and CVD by middle age, suggesting that any CAC is abnormal in this age range. Compared with the CARDIA study, we found a higher prevalence of CAC (34.4% vs 10%), likely owing to the presence of clinical indications for CAC scoring and therefore a high prevalence of elevated lifetime risk for CVD as opposed to a general community representative sample. However, in comparison with the hazard ratios for incident CHD and CVD events in the CARDIA study,^14^ we found a comparable 5-fold higher likelihood of CHD mortality and 3-fold higher likelihood of CVD mortality in those with elevated CAC.

### Determinants of Premature CAC

Although age is the dominant factor associated with the development of CAC, multiple traditional risk factors and lifestyle behaviors have been associated with the development of premature CAC. Prior data from CARDIA demonstrated that baseline level of low-density lipoprotein cholesterol, cigarette smoking, systolic blood pressure, and blood glucose level in participants at baseline (aged 18-30 years) all were independently correlated with the likelihood of subsequent CAC 15 years later.^21^ In addition, higher intake of fruits and vegetables, high levels of cardiorespiratory fitness, and lack of abdominal obesity have been associated with a lower likelihood of premature CAC.^22,23,24^

Accordingly, our data demonstrated a higher likelihood of CAC across all traditional CVD risk factors, and the prevalence of CAC increased as the burden of risk factors increased. Despite this finding, more than 40% of those with a CAC score higher than 100 had 0 or 1 reported CVD risk factor. More important, prior research has shown that individuals with CAC but no traditional CVD risk factors are at significantly elevated risk for CHD events compared with those with traditional risk factors but no CAC.^25^

### CAC Testing Among Younger Adults

The recent 2018 cholesterol guidelines from the American Heart Association and the American College of Cardiology recommend CAC scoring for intermediate-risk (10-year CVD risk, ≥7.5% to <20%) individuals who are uncertain about their decision to start statin therapy.^26^ Prior analyses have suggested that, for individuals who are uncertain about statin use, CAC testing may be the best option to determine the magnitude of the potential benefit from statin therapy.^9,10^ In addition, CAC scoring has been proposed to help determine an individual’s likelihood of benefit from aspirin as well as antihypertensive therapy.^11,12^

Our data suggest that the clinical utility of CAC testing may extend to select younger adults, mainly those at elevated lifetime risk for CVD and uncertainty regarding treatment decisions. Even for individuals in their 30s, despite their young age, approximately 1 in 5 had CAC. Women were less likely to have CAC; therefore, CAC scoring for young women should be considered cautiously given the low likelihood of a positive finding. However, given the exponential nature of CAC progression,^14^ the presumed high lifetime risk of individuals with CAC, and the general safety and inexpensive nature of most preventive medications, considering tailored preventive pharmacotherapy in the context of a clinician-patient risk discussion appears reasonable for young men and women with any CAC.

A CAC score of 0 has been repeatedly shown to be associated with very low risk for CHD and CVD events.^27,28^ For young adults at elevated lifetime risk, a CAC score of 0 does not lessen the importance of heart-healthy lifestyle behaviors, but given that many patients would prefer to avoid preventive medications, the potential for CAC testing to identify patients for whom lifestyle therapies can be favored instead of preventive medications remains an important patient-centered outcome.

### Strengths and Limitations

Our analysis benefited from data from multiple experienced centers geographically dispersed throughout the United States with a large sample size of both men and women with long-term follow-up. In addition, the ability to analyze the association of CAC with cause-specific mortality during 12 years of follow-up is novel, and the large sample size allowed subgroup analyses by age, sex, and lifetime risk.

Referral bias is a potential limitation, and generalizability must be interpreted in the appropriate context. The exact clinical indications for CAC scoring were not documented, although most participants underwent CAC scoring owing to the presence of a major CVD risk factor (mainly hyperlipidemia or a family history of CVD) or multiple CVD risk factors. However, as opposed to general screening, most experts recommend CAC testing as a tool for clinical decision-making as part of a sequential process of risk assessment.^29^

Therefore, our results, which represent the prevalence and outcomes associated with CAC among younger individuals with clinical indications for CAC scoring, remain clinically relevant. There were individuals in our analysis with missing data, but most were missing only 1 risk factor, and there was excellent correlation between mean and median ASCVD risk scores calculated directly compared with scores calculated using imputed data.

Other limitations include self-reporting of risk factors and the inadequacies of vital status ascertainment in the United States, with a potential for up to 10% underestimation of mortality,^30^ although this underestimation would be expected to be nondifferential across CAC groups. We used the PCE to get a general sense of the estimated 10-year cardiovascular risk in our sample, although the American Heart Association and American College of Cardiology guidelines are clear that there are no data to support the use of the PCE for individuals younger than 40 years. Also, 87.7% of our study sample was of white race/ethnicity, and we were unable to examine associations specifically among nonwhite races/ethnicities.

## Conclusions

In a large, multicenter cohort of young adults with long-term follow-up, CAC was not uncommon, with elevated CAC scores independently associated with higher rates of CHD, CVD, and all-cause mortality. For younger individuals with low 10-year risk of ASCVD but with other risk indicators, CAC scoring may provide a method for further risk assessment to help guide the intensity of preventive interventions.

## Notes

Supplement. **eTable.** Subdistribution Hazard Ratios for CHD and CVD Death and Hazard Ratios for All-Cause Mortality According to CAC in 22 346 Individuals age 30-49 Years Stratified by Lifetime Risk of CVD **eFigure.** The Prevalence of CAC According to Age Stratified by Sex
