# Supplementary material for: Association of Coronary Artery Calcium With Long-term, Cause-Specific Mortality Among Young Adults
Source: JAMA Netw Open. 2019 Jul 19;2(7):e197440. doi: 10.1001/jamanetworkopen.2019.7440 (PMC6646982; doi:10.1001/jamanetworkopen.2019.7440)
Supplement: Supplement. — eTable. Subdistribution Hazard Ratios for CHD and CVD Death and Hazard Ratios for All-Cause Mortality According to CAC in 22 346 Individuals age 30-49 Years Stratified by Lifetime Risk of CVD eFigure. The Prevalence of CAC According to Age Stratified by Sex [file jamanetwopen-2-e197440-s001.pdf]

Supplementary Online Content

Miedema MD, Dardari ZA, Nasir K, et al. Association of coronary artery calcium with long-term, cause-specific mortality among young adults. *JAMA Netw Open.* 2019;2(7):e197440. doi:10.1001/jamanetworkopen.2019.7440

eTable. Subdistribution Hazard Ratios for CHD and CVD Death and Hazard Ratios for All-Cause Mortality According to CAC in 22 346 Individuals age 30-49 Years Stratified by Lifetime Risk of CVD
eFigure. The Prevalence of CAC According to Age Stratified by Sex

This supplementary material has been provided by the authors to give readers additional information about their work.

© 2019 Miedema MD et al. *JAMA Network Open.*

eTable. Subdistribution Hazard Ratios for CHD and CVD Death and Hazard Ratios for All-Cause Mortality According to CAC in 22 346 Individuals age 30-49 Years Stratified by Lifetime Risk of CVD

**Low Lifetime Risk (n=8,113)**	Unadjusted	Model 1	Model 2
CAC Prevalence*	**CHD Death**		
CAC 0	1.0	1.0	1.0
CAC 1-100	4.8 (0.79, 28.7)	3.1 (0.46, 20.5)	2.9 (0.44, 19.3)
CAC >100	8.1 (0.74, 89.0)	4.2 (0.28, 62.6)	3.7 (0.25, 55.4)
**CVD Death**			
CAC 0	1.0	1.0	1.0
CAC 1-100	2.7 (0.91, 8.1)	2.2 (0.69, 6.8)	2.2 (0.69, 6.8)
CAC >100	7.0 (1.8, 27.2)	5.1 (1.1, 23.1)	5.0 (1.1, 23.4)
**All Cause Death**			
CAC 0	1.0	1.0	1.0
CAC 1-100	1.4 (0.79, 2.4)	1.1 (0.64, 2.0)	1.1 (0.64, 2.0)
CAC >100	3.2 (1.5, 6.7)	2.3 (1.1, 5.0)	2.3 (1.04, 5.0)
**High Lifetime Risk (n=14,233)**			
CAC Prevalence*	Unadjusted	Model 1	Model 2
**CHD Death**			
CAC 0	1.0	1.0	1.0
CAC 1-100	1.8 (0.78, 4.3)	1.7 (0.68, 4.0)	1.5 (0.61, 3.7)
CAC >100	8.2 (3.7, 18.3)	7.3 (3.1, 17.5)	5.8 (2.5, 13.6)
**CVD Death**			
CAC 0	1.0	1.0	1.0
CAC 1-100	1.5 (0.88, 2.7)	1.5 (0.83, 2.7)	1.3 (0.75, 2.4)
CAC >100	4.1 (2.2, 7.4)	3.9 (2.0, 7.6)	3.1 (1.6, 5.9)
**All Cause Death**			
CAC 0	1.0	1.0	1.0
CAC 1-100	1.3 (0.98, 1.8)	1.3 (0.98, 1.8)	1.3 (0.93, 1.7)
CAC >100	3.2 (2.3, 4.4)	3.1 (2.2, 4.4)	2.7 (1.9, 3.9)

* CAC Prevalence categorized according to Agatston scores of zero, 1-100, and >100
Hazard ratios (95% Confidence Intervals) are presented with 1.0 indicating the reference value
Abbreviations: CHD – Coronary Heart Disease; CVD – Cardiovascular Disease, CAC – Coronary Artery Calcium
Model 1 is adjusted for age and sex
Model 2 is adjusted for age, sex, hyperlipidemia, hypertension, smoking, diabetes, and a family history of CHD.

© 2019 Miedema MD et al. *JAMA Network Open.*

eFigure. The Prevalence of CAC According to Age Stratified by Sex

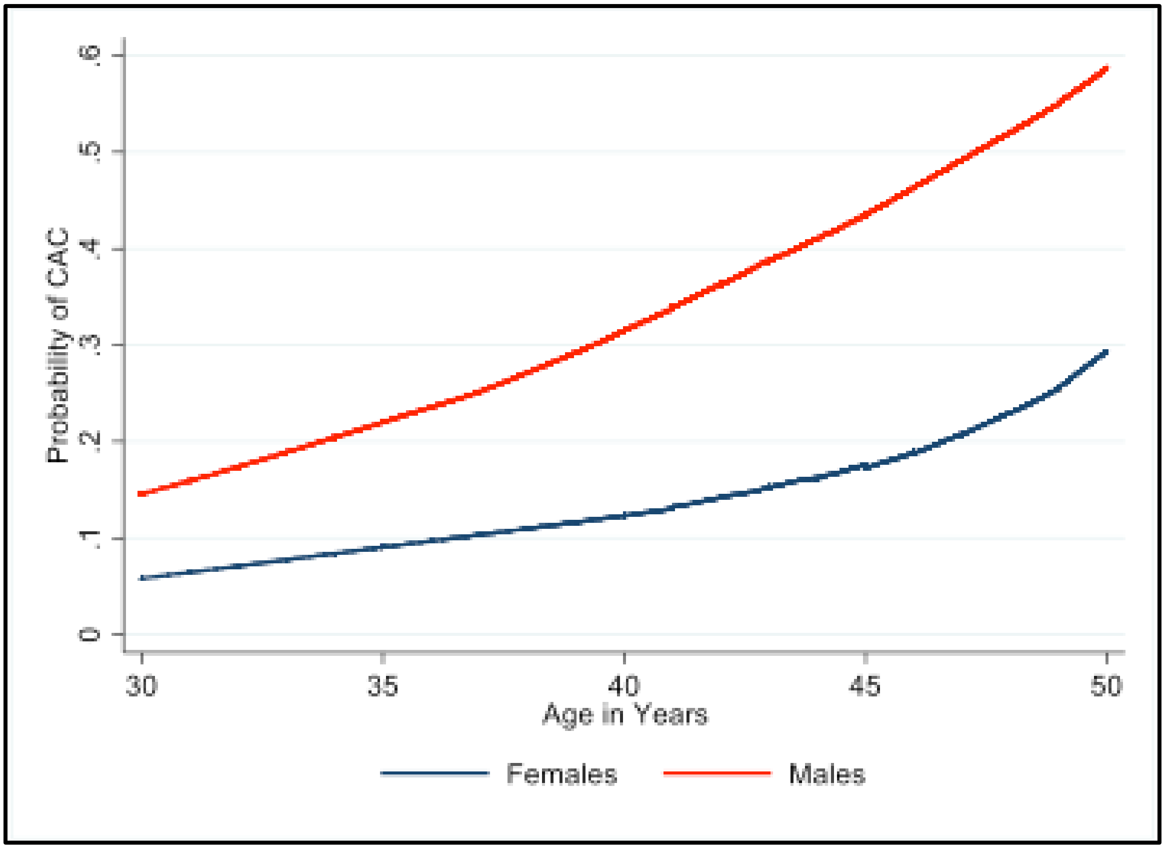

Figure Legend. The prevalence of CAC according to age and sex in 22,346 individuals 30-49 years old in the CAC consortium.

Abbreviation: Coronary Artery Calcium – CAC

© 2019 Miedema MD et al. *JAMA Network Open.*
